# Eat Me If You Can: Cognitive Mechanisms Underlying the Distance Effect

**DOI:** 10.1371/journal.pone.0084643

**Published:** 2013-12-18

**Authors:** Astrid F. Junghans, Catharine Evers, Denise T. D. De Ridder

**Affiliations:** Department of Clinical and Health Psychology, Utrecht University, The Netherlands; University G. d'Annunzio, Italy

## Abstract

Proximal objects provide affordances that activate the motor information involved in interacting with the objects. This effect has previously been shown for artifacts but not for natural objects, such as food. This study examined whether the sight of proximal food, compared to distant food activates eating-related information. In two experiments reaction times to verbal labels following the sight of proximal and distant objects (food and toys) were measured. Verbal labels included function words that were compatible with one object category (eating and playing) and observation words compatible with both object categories. The sight of food was expected to activate eating-related information when presented at proximity but not at distance, as reflected by faster reaction times to proximal than distant compatible eating words and no difference between reaction times to proximal and distant food for observation words (Experiment 1). Experiment 2 additionally compared the reaction times to wrapped and unwrapped food. The distance effect was expected to occur only for unwrapped food because only unwrapped food is readily edible. As expected, Experiment 1 and 2 revealed faster responses to compatible eating words at proximity than at distance. In Experiment 2 this distance effect occurred only for readily edible, unwrapped food but not for wrapped food. For observation words no difference in response times between the distances was found. These findings suggest that the sight of proximal food activates eating-related information, which could explain people’s differential behavioral responses to reachable versus distant food. The activation of eating-related information upon sight of accessible food could provide a cognition-based explanation for mindless eating.

## Introduction

 The current escalation of problematic eating behaviors and obesity is commonly explained by people’s tendency to overeat and choose unhealthy food. This tendency is enhanced by the constant provision of food in today’s environment, which people often cannot resist. Consumption is exceptionally high when food is located within people’s physical reach [1]. This observation provides the foundation for the current research, which aims at investigating the underlying mechanism for the increased consumption of food within people’s reach. Specifically, the current research investigates whether the accessibility of food modulates the activation of eating-related information. 

 To answer this question a paradigm from cognitive psychology is applied to assess whether accessible food activates an eating affordance that may stimulate eating behavior. Affordances are potential interactions between an object and its perceiver. Objects afford interactions (e.g. grasping, reaching, sitting) and thereby activate the motor information involved in interacting with the object [2–5]. Past research has repeatedly shown the activation of motor information upon sight of proximal objects [6]. For example, Costantini and colleagues [7] employed the spatial alignment paradigm to show that interaction-relevant object features evoke motor activations. Perceiving the handle of a cup to the right or left side facilitates responses with the right or left hand, respectively. However, these studies have focused on artificial rather than natural objects because of their constitutive functionality. Yet, it seems likely that motor information is equally activated upon the sight of natural objects, such as food, under the condition that those are relevant to the observer. However, hitherto this has not been examined empirically.

 Therefore, this study employs a reaction time measure to assess the activation of information related to potential interaction at the sight of natural and artificial objects; The activation of eating information upon sight of accessible food and the activation of playing information upon the sight of accessible toys. This information is expected to be activated due to the affordances exerted by the actionable objects. This comparison should clarify the differences and similarities between affordances posed by natural and artificial objects. Furthermore, applying the concept of affordances to understanding eating behavior will enhance health psychologists’ knowledge of the mechanisms instigating mindless eating behavior and, eventually, enrich the field with novel opportunities to support people in their attempts to eat less and more healthily. 

## Theoretical Background

 People consume more food when it is located in their proximity than at a distance [8]. This effect can be observed in distances as small as 50 cm. Placing food at 20 cm distance compared to 70 or 140 cm distance has shown to significantly increase consumption [1]. While effort has often been suggested as the underlying mechanism of this distance effect [9], the current research examines whether the difference in consumption is, at least partially, driven by the eating affordances posed by proximal food.

 The difference between proximity (within arm’s reach) and distance (out of arm’s reach) can be more clearly defined by the concepts of peripersonal and extrapersonal space. Whereas the peripersonal space refers to the area within reach, the extrapersonal space lies outside of reach. The precise difference is constituted by a person’s movement-related spatial map. Neurons in the ventral premotor cortex (area F4) are activated by visual and tactile receptors in such way that the visual receptive field is defined by the tactile receptive field and the area immediately adjacent to it [10]. Moreover, these neurons respond to objects located within the peripersonal space, thereby determining whether objects activate motor information or not [10]. Consequently, the terms proximity and distance, as used in the current research, are defined by the peripersonal and extrapersonal space. 

 Objects within this proximity provide immediate potential interactions, so-called affordances that activate information required for the interaction [7,11]. The concept of affordances [12] stems from the notion that observing an object facilitates the possible interactions with the object [13]. Behavioral studies have shown that the mere perception of (images of) objects activates the motor-acts required for the interaction with the objects. Thus, observing a glass activates the movement involved in reaching for the glass. Similarly, handles afford to be grasped, chairs afford to be sit on, and stairs afford to be climbed [3,6,7,14,15]. 

 Whether objects present immediate affordances depends, on the one hand, on the spatial location of the objects. Objects generally activate motor information when they are located within reach [16,17]. On the other hand, affordances depend on the functionality of the objects. Functionality refers to the possibility to use the objects for their specific function. This implies that objects only activate motor information if they are functional. If objects are presented in such way that prevents their usage, no motor information is activated. Perceiving a bottle of water and a full glass has shown not to activate the affordance to pour as the glass is already full and therefore not functional [4]. Both of these requirements appear to be at odds with the idea of affordances posed by *images* of objects as those are neither reachable nor functional: They are, as a matter of fact, not actionable [18]. Yet, what matters for the activation of motor information is not the realistic actionability, but the *perceived* actionability. Objects do not require to be realistically reachable and functional, but to lie within a spatial location that is perceived as reachable and in a manner in which the object appears functional. Therefore, images of functional objects that appear to be within reach should activate object-specific motor information despite their inactionability [7,19].

 It has previously been argued that artifacts, such as cups, provide stronger affordances than natural objects, such as food, because they are specifically designed for a particular function and are thus mentally represented in terms of this functionality. Natural objects, on the other hand, are supposedly represented mentally more in terms of their sensory properties such as color, shape, and size [19]. After all, natural objects have not deliberately been designed for a particular function; instead, existing natural objects are made use of when people can use them to fulfill a function. Food has originally not been designed for eating, and yet, people use it to satisfy their hunger. Despite the fact that today’s food is often processed rather than naturally grown, it maintains the quality of possessing an inartificial, natural function as opposed to artifacts that fulfill more designated functions. Consequently, it is proposed that relevant natural objects, that are used to serve a function, should also exert affordances. Considering its evolutionary importance for survival, food represents a highly relevant natural object that functions as energy source [20,21]. Therefore, food should present affordances in a similar manner as artifacts. 

 The above presented theorizing based on grounded cognition shows how cognitive processes are dependent on the environment in which they occur. Whether objects exert affordances depends on the potential to interact with the objects. Only functional and accessible objects exert affordances and thereby activate the information related to the specific interaction. 

 Grounded cognition research has also provided evidence showing that language comprehension and motor activations are neurologically linked [22]. Previous research has, for example, shown that evaluating grammatical correctness of sentences involving supposedly irrelevant directions (away and toward) facilitated responses requiring bodily movement compatible to the movement of the actions implied by the meaning of those sentences [23]. Consequently, objects should activate the information related to potential interaction with the objects both in terms of motoric activation and verbal information. On the one hand, observing an object should activate its semantic concept, thereby causing a readiness to respond to words compatible with the object. Seeing food should lead to an activation of the food and eating concept, thereby leading to faster recognition of food and eating related words. On the other hand, observing a proximal object should activate the motoric response involved in interacting with the object, thereby causing a readiness to move the muscles involved in the afforded action. Consequently, observing proximal food should afford grasping for the food as that is the first step involved in eating thereby activating the muscles involved in the grasping movement. This muscular readiness not only facilitates motoric responses to the actually afforded action, the grasping, but also other actions that rely on the same muscles as corroborated by Wilf, Holmes, Schwartz, and Making [24]. 

 Due to their neurological link semantic and motoric activations cannot be disentangled using the current paradigm. Consequently, potentiated responses could be explained by the activation of either semantic or motoric activation. Nevertheless, as presented above, research has repeatedly shown the modulation of motoric, rather than semantic activation by distance and accessibility. Therefore, potentiated responses are expected to be driven by motoric activations rather than semantic activations. Nevertheless, the terms eating-, and playing-related information will be employed to refer to both semantic and motoric activations. 

 As has been done previously [7,17], this research uses reaction times to verbal labels to measure what action-related information is activated upon exposure to objects. The activation of eating- and playing-related information is therefore a measure of whether the potential to play or eat is activated upon the sight of accessible and inaccessible toys and food, respectively. 

### The present research

 Based on this rationale, the current research examines whether accessible, as opposed to inaccessible food (natural object), and toys (artifacts), activate the information related to eating or playing, respectively. The activation of this information is measured using reaction times to verbal labels that represent object-compatible (e.g. food and eating) or object-incompatible (e.g. toy and eating) interactions. Participants have to judge the compatibility of function and observation words with proximally and distally presented objects. Function words are related to the interaction with the objects. Compatible function pairs are therefore foods and eating words as well as toys and playing words. Observation words are compatible with both food and toys. 

 We hypothesize that reaction times to compatible function words are faster when they follow the sight of a proximal rather than a distant object. Only proximal food and toys are expected to activate eating- and playing-related information, respectively, because only proximal food and toys allow for immediate interaction. Consequently, seeing proximal rather than distant food should activate eating-related information: On the one hand it should lead to an activation of the semantic concept of food leading to faster recognition of eating related words. On the other hand it should potentiate the muscles involved in grasping for the food (and involved in pushing the response button), which should lead to faster reaction times to eating words following food within reach than food outside of reach. Analogously, seeing a proximal rather than distant toy should activate playing-related information, which should lead to faster reaction times to playing words following reachable toys compared to not reachable toys. Since the observation of objects is possible for both proximal and distant objects, reaction times to observation words should not depend on distance. Therefore, there should be no difference in reaction times between responses to observation words following proximal and distant objects. Furthermore, we examined whether motivational states, such as hunger influence the activation of eating-related information, as the relevance of food potentially depends on such motivational states. After all, hungry people could experience stronger activations of eating-related information when perceiving proximal food than satiated people. To our knowledge, such motivational influences on the nature of information activation and relatedly on affordances have not been investigated to this date. 

 Two experiments were conducted to examine these hypotheses. Experiment 1 investigated the activation of eating- and playing-related information by objects at proximity and distance in general, as well as the activation of eating- and playing-related information by toys and food independently, whereas Experiment 2 extended the range of food to include both wrapped and unwrapped food. Wrapped food cannot readily be eaten. It is not actionable. Consequently, eating-related information should not be activated upon the sight of proximal wrapped food. Unwrapped food, on the other hand, can readily be eaten, therefore is actionable, and should activate eating-related information. This additional manipulation extends the factor of accessibility to not only include the effect of distance on the activation of eating-related information but also the effect of packaging. 

 Investigating these three kinds of objects (toys, wrapped food, unwrapped food) will shed light on the differences and similarities of their respective activations of eating- and playing-related information. It will reveal whether natural objects activate this information in the same manner as artifacts, and whether the perceived possibility to readily interact with the object influences the nature of these activations. Furthermore, examining the activation of eating-related information will deepen health psychologists’ understanding of the influences of unconscious, cognitive processes involved in the representation of food. If food is represented differently depending on context and affords eating only when it is accessible, then behaviorally observed responses to food in the environment can be better understood, explained, and eventually circumvented. 

## Experiment 1

 The experiment entailed a 2 (object: food vs. toys) x 2 (distance: proximity vs. distance) x 2 (word: observation vs. function) factorial design. It was firstly hypothesized that participants react faster to function words following proximal compatible objects than distant compatible objects. This distance effect should not occur for observation words. More specifically, the second hypothesis predicted faster reaction times to eating words following food at proximity than following food at distance. Similarly, participants should react faster to playing words following toys at proximity compared to toys at distance. Reaction times to observation words should be similar at both distances. Finally, stronger motivation to obtain food, due to hunger, was expected to be positively associated with the distance effect for food and eating.

## Methods

### Sample and participant selection

 Participants (*N* = 52) were recruited from the campus of Utrecht University to take part in this experiment for money (4 Euro) or course credit. All participants were native Dutch speakers and had normal or corrected to normal vision, with the exception of four participants who reported to require correction but not wearing any during the experiment. Since excluding these participants from the analysis led to the same results they remained included. Participants (*N* = 5) not responding to 50% or more of the compatible observation trials were excluded for not adhering to the task. The final dataset included 47 participants (33 women, 14 men) with an average age of 20.34 (SD = 1.97), and an average BMI of 21.77 (SD = 2.3). 

 The study was conducted in accordance with the ethical standards described by the Medial Research Involving Human Subject Act [25]. This Act exempts research on healthy human subjects from review for as long as it does not involve any invasion of participants’ integrity. Consequently, no formal ethical approach was required according to Dutch national standards. Written consent was required from each participant prior to participation. 

### Procedure

 Participants were seated in front of a computer screen at a distance of approximately 48 cm. Before starting the actual experiment participants completed eleven practice trials in which they received feedback on the correctness of their response to ensure that participants understood the concept of compatibility between words and images. The actual experiment consisted of 216 trials in which participants were first exposed to an image of food or toys for one second, followed by a word (eating, playing, or observation). The word was presented on the area of the screen that was on average at equal distance from the proximal and the distant object on the image. Participants had to decide whether the word was compatible with the object by clicking the space bar or whether it was incompatible with the object by refraining from any response. The word remained on the screen until the participant had responded or 2.5 seconds had passed. Each participant was exposed to each combination of image and word exactly once at random order. Thus, 33% of the trials were incompatible (toy and eating word; food and playing word). After the experimental procedure participants filled in a questionnaire assessing demographic variables and level of hunger. Finally, they were debriefed, thanked, and granted their rewards. 

### Materials

####  Images

Images of food and toys were presented on computer screens. The images were photographs on which the target object was located on the near or far end of a table elongated into the distance. Metrically, the proximal object was at ~ 50 cm (within perceived reach) and the distant object at ~ 180 cm distance (outside perceived reach) from the viewpoint of the photographer. 

 The perceived distances of the objects in the images were pre-tested as part of a larger study. Participants (*N* = 95) rated whether objects at either distance were reachable or not (1 = reachable; 2 = not reachable). A Wilcoxon signed-rank test revealed that all proximal objects (all Mdns = 1) were rated as reachable significantly more often than distant objects (all Mdns = 2), - all *p*s < .001 -. The final set of objects on the food pictures, all presented in glass bowls, included M&Ms, apples, muffins, carrots, cookies, and chips. These foods were chosen to include different levels of healthiness, salty and sweet choices, as well as a wide range of different colors. The images of toys included building blocks, a puzzle, and different kinds of construction sets.

####  Verbal labels

Words were selected on the basis of compatibility with images of food and toys. In an online pre-test native Dutch participants (*N* = 25) were asked to rate the degree of compatibility (0 = low compatibility - 100 = high compatibility) between six eating words, six playing words, and six observation words with nine food images and six toy images (6 of these food images were later selected for the main study based on perceptibility, variability of food, and coherence with previous studies). The three eating and playing words most compatible with images of food and toys, respectively, were chosen for the study (toys: to play (*M* = 85.5; *SD* = 20.47), to build (*M* = 85.3; *SD* = 13.66), to assemble (*M* = 82; *SD* = 17); food: to eat (*M* = 87.3; *SD* = 15.97), to consume (*M* = 83.4; *SD* = 11.86), to taste (*M* = 80.4; *SD* = 12.41)). A Wilcoxon signed-rank test revealed that each of these words was significantly more compatible with its object category than the other object category (all *p*s < .001). The three most compatible observation words were chosen on the basis of compatibility with all the objects (to see (*M* = 54.6; *SD* = 17.6), to watch (*M* = 50.9; *SD* = 16.87), to perceive (*M* = 51.9; *SD* = 17.36)). Note must be taken of a significant difference in compatibility between food and toys with observation words. Observation words were more compatible with toys (*Mdn* = 61) than with food (*Mdn* = 48) (*z* = -3.23; *p* = .001). This difference has to be taken into account in the analysis of the main studies.

### Measures

 Reaction time mean scores for each combination of compatible object and word category for proximity and distance were computed and natural log transformed to normalize the distribution. Furthermore, +/- 2 *SD* from the mean of the respective category were excluded from the analysis. This exclusion led to different numbers of participants being excluded from the different analyses. For the ease of interpretation means will be reported in reaction times (milliseconds). Error rates, representing the lack of responses to compatible pairs, were calculated for each category and ranged from 2,7% to 16,2%. 

 In order to examine whether motivation influences the activation of eating-related information, level of hunger (1 = not hungry – 3 = very hungry) was assessed with one item. 

## Results

### General Affordance Activation

 To test the hypothesis that participants respond faster to compatible function words following proximal than distant objects in general a repeated measures ANOVA was conducted with object (toys vs. food), distance (proximity vs. distance) and word (compatible function vs. observation) as within-subject-factors. The results revealed a significant main effect of object, *F*(1,38) = 24.105; *p* <.001; *η* ^2^_*p*_ = .388, with participants responding faster to food (*M* = 796.16; *SD* = 91.27) than toys (*M* = 839.62: *SD* = 117.77); a significant main effect of word, *F*(1,38) = 46.842; *p* < .001; *η* ^2^_*p*_ = .552, with participants responding faster to compatible function words (*M* = 779.05; *SD* = 87.83) than observation words (*M* = 856.73; *SD* = 105.78); and a significant interaction between object and word, *F*(1,38) = 35.956; *p* < .001; *η* ^2^_*p*_ = .486. For compatible function words participants responded significantly faster to food (*M* = 736.3; *SD* = 80.67) than to toys (*M* = 821.8; *SD* = 107.83), *F*(1,38) = 52,585; *p* < .001; *η* ^2^_*p*_ = .581. However, there was no significant difference between reaction times to observation words between toys (*M* = 857.44; *SD* = 138.38) and food (*M* = 856.02; *SD* = 102.72), *F*(1,38) = 0.0; *p* = .99. There was no significant main effect of distance *F*(1,38) = 2.214; *p* = .15. Finally, a significant interaction between distance and word was found, *F*(1,38) = 8,649; *p* < .01; *η* ^2^_*p*_ =.185. Participants responded faster to function words following compatible objects at proximity (*M* = 760.52; *SD* = 82.44) than at distance (*M* = 797.57; *SD* = 102.33), *F*(1,38) = 15.635; *p* < .001; *η* ^2^_*p*_ = .292. For observation words there was no significant difference between reaction times to proximal (*M* = 863.76; *SD* = 135.72) and distant objects (*M* = 849.7; *SD* = 129.09), *F*(1,38) = 1.085; *p* = .3. 

### Food

**Figure 1:**
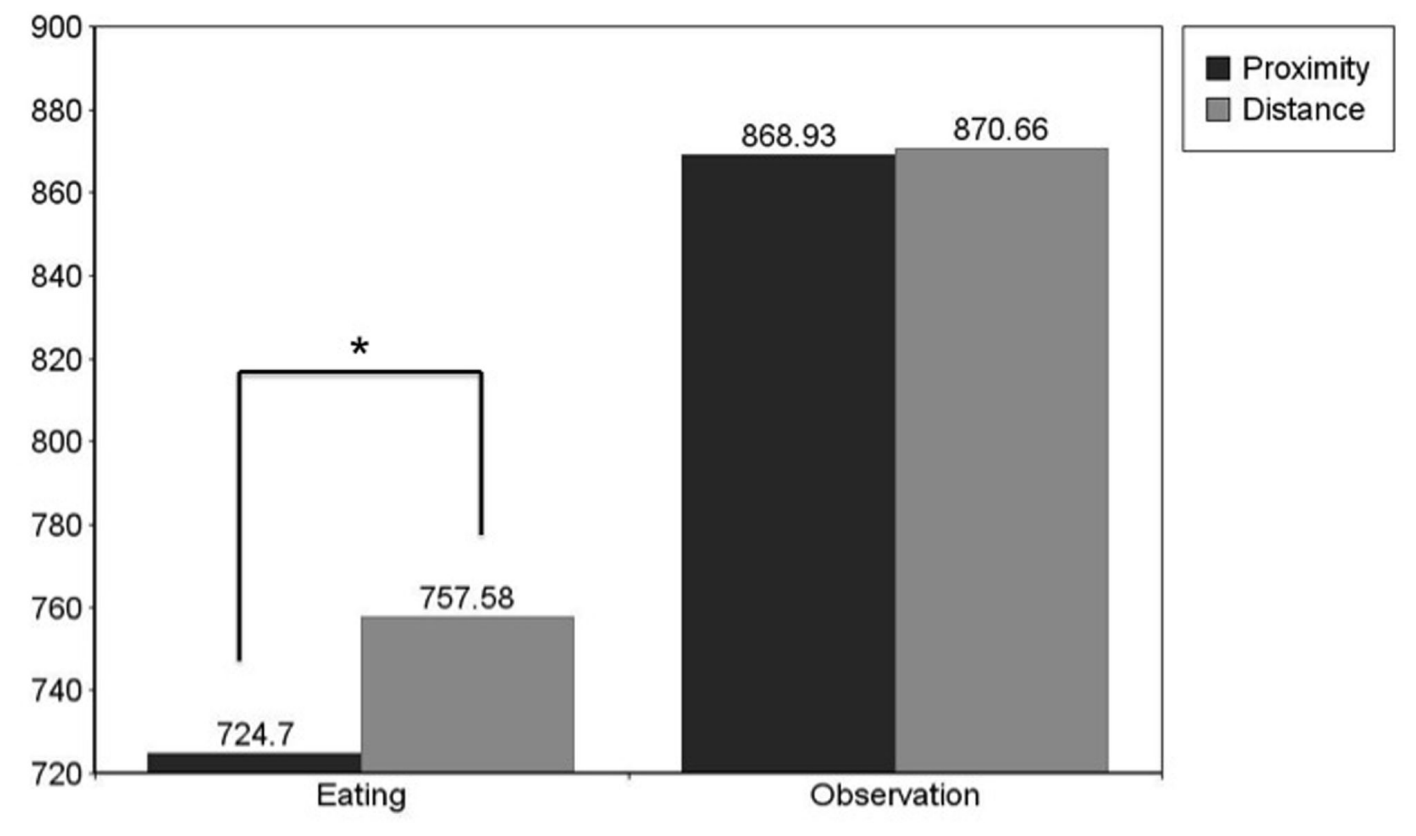
Experiment 1. **Mean Reaction Times to Food**. Mean reaction times to eating and observation words following food images at proximity and at distance: Reaction times to proximal eating words are significantly faster than reaction times to distant eating words p < .05.

 To test the distance effect specific to food planned contrasts were conducted. Paired samples t-tests revealed that participants responded significantly faster to eating words following proximal food (*M* = 724.7; *SD* = 85.09) than distant food (*M* = 757.58; *SD* = 94.99), *t*(43) = -3.341; *p* (2-tailed) < .01; Cohen’s d = .36. For observation words no significant distance effect was found between proximal (*M* = 868.93; *SD* = 168.85) and distant (*M* = 870.66; *SD* = 143.11) objects, *t*(43) = -0.283; *p* = .78 (See Figure 1.). 

### Toys

**Figure 2:**
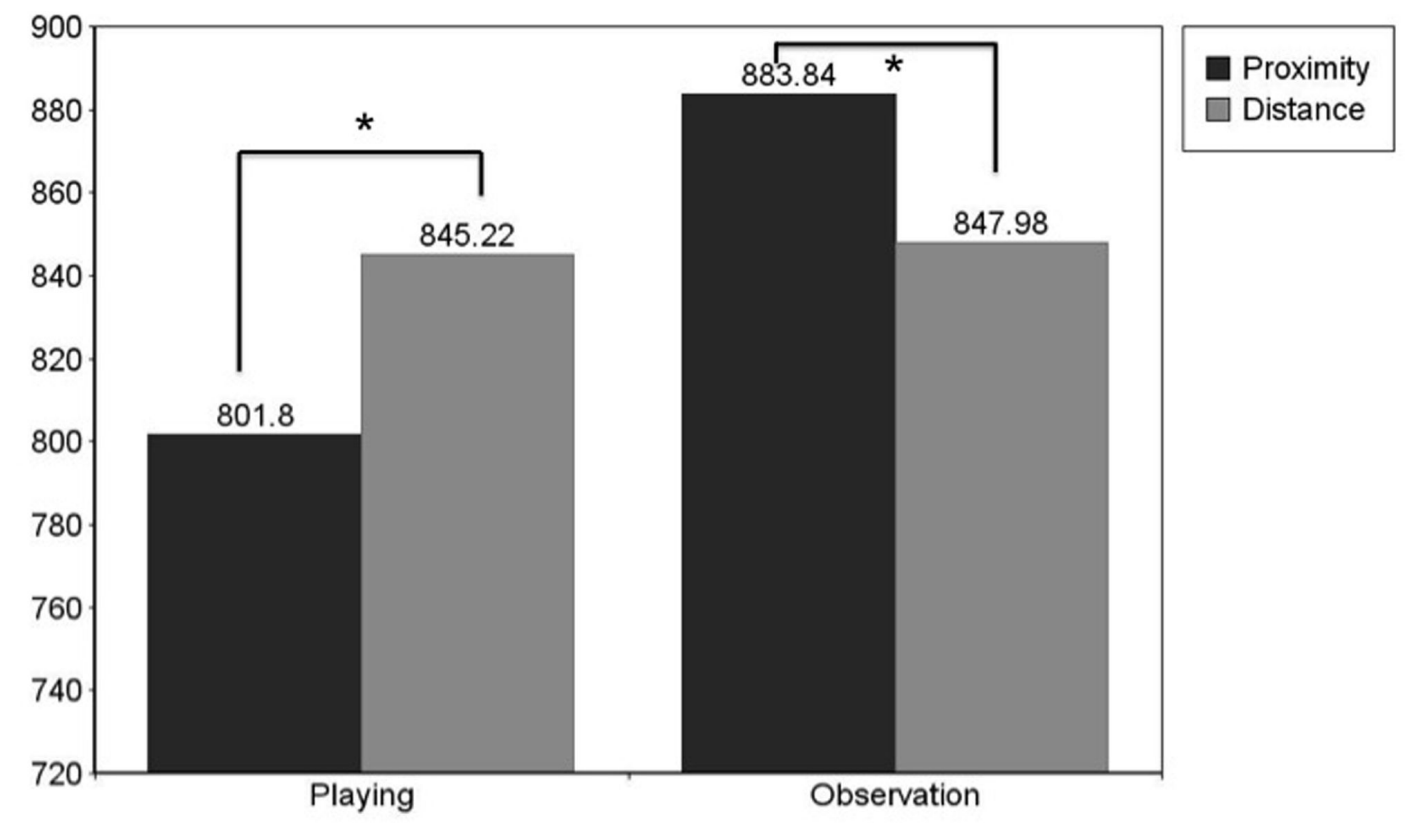
Experiment 1. **Mean Reaction Times to Toys**. Mean reaction times to playing and observation words following toy images at proximity and at distance: Reaction times to proximal playing words are significantly faster than reaction times to distant playing words p < .05. Reaction times to distant observation words are significantly faster than reaction times to proximal observation words p < .05.

 To test the distance effect specific to toys similar planned contrasts were run. The paired samples t-tests revealed significantly faster reaction times to playing words following toys at proximity (*M* = 801.8; *SD* = 104.3) than at distance (*M* = 845.22; *SD* = 135.85), *t*(43) = -2.791; *p* (2-tailed) < .01; Cohen’s d = .36. For observation words participants responded significantly faster following distant toys (*M* = 847.98; *SD* = 162.72) than proximal toys (*M* = 883.84; *SD* = 161.12), *t*(41) = 2.482; *p* = .02; Cohen’s d = .22 (See Figure 2.)

### Hunger

 Level of hunger was not significantly associated with the reaction time difference score (*r* = -.06; *p* = .689). This difference score (difference between reaction times to eating words and food at proximity vs. distance) was computed to represent the strength of the distance effect. 

## Discussion

 In support of the first hypothesis participants responded faster to compatible function words following proximal than distant objects. This implies that images of proximal food and toys activate eating- and playing-related information more strongly than distant objects, indicating the necessity of potential interaction in terms of spatial location for the activation of this information. Additionally, the results revealed faster responses to food in general which could be explained by the strong evolutionary relevance of food objects. Participants also responded generally faster to compatible function words than to observation words regardless of distance as shown by the main effect of word. 

 Planned contrasts revealed that eating-related information was activated upon the sight of proximal but not distant food. No distance effect was found for observation words. This finding indicates that eating-related information was activated and consequently responded to faster when interaction was possible due to the spatial location of the food. Only proximal food provides the potential to eat, leading to differential reaction times to eating words between the two distances. At the same time both proximal and distant food provide the potential to be looked at, leading to no differential reaction times to observation words following proximal and distant objects. 

 For toys the planned contrasts supported the activation of playing-related information upon the sight of proximal toys compared to distant toys. Surprisingly, a reverse distance effect was found for observation words with participants responding faster to observation words following toys at distance than at proximity. This finding cannot be explained at this point but Experiment 2 aimed at replicating these findings to unveil whether this reverse distance effect is stable and represents a structural difference between the information activation related to potential interaction posed by natural and artificial objects. 

 Level of hunger was not significantly related to the distance effect on food and eating words. Thus, the activation of eating-related information seemed unaffected by the motivational state of the perceiver. This implies that the motivational state of the perceiver does not influence the affordances exerted by objects. Experiment 2 included additional motivational measures to examine whether this lack of motivational influence is consistent across different motivational states.

 Although Experiment 1 provided initial support for the activation of eating-related information exerted by proximal food Experiment 2 aimed at investigating the influence of the accessibility of food in more detail. In addition to examining the accessibility due to distance Experiment 2 included the accessibility due to packaging. More specifically, it was tested whether the packaging of food similarly modulates the activation of eating-related information. Wrapped food cannot readily be eaten and should consequently not exert the affordance to eat. Unwrapped food, on the other hand, can readily be eaten and should activate the affordance to eat. To test this, participants were presented with images of wrapped and unwrapped food. It was ensured that each food package was transparent so that the food itself remained visible. 

## Experiment 2

 The experiment was based on a 2 (object: food vs. toys) x 2 (distance: proximity vs. distance) x 2 (word: eating vs. observation) x 2 (packaging: wrapped vs. unwrapped; only for food) within-subject design. Similarly to Experiment 1 we hypothesized that participants respond faster to function words following proximal compatible objects than distant compatible objects in general and that this distance effect does not occur for observation words. Secondly, we expected the distance effect for food to occur only upon the sight of unwrapped but not wrapped food. Consequently, participants should be faster responding to eating words following proximal unwrapped food compared to distant unwrapped food. This distance effect should occur neither for wrapped food nor for observation words. Thirdly, stronger motivation to obtain food was expected to be positively associated with the distance effect for unwrapped food and eating. Finally, faster reaction times to playing words following proximal toys than distant toys and no such effect for observation words were expected. 

## Methods

### Sample and Participant Selection

 Participants (*N* = 71) were recruited from the campus of Utrecht University for course credit or money (4 Euro). All participants were native Dutch speakers and had normal or corrected to normal vision. Some participants (*N* = 3) reported to require correction but not wearing any during the experiment. Since excluding these participants from the analyses led to the same results they were retained. Additionally, participants (*N* = 8) not responding to 50% or more of the compatible observation trials were excluded from the analysis for not adhering to the task. The final dataset included 63 participants (49 women, 13 men, 1 unknown) with an average age of 21.21 (SD = 3.31), and an average BMI of 21.35 (SD = 2.55).

 As previously, the study was conducted in accordance with the ethical standards described by the WMO. Written consent was required from each participant prior to participation. 

### Procedure & Materials

 The procedure and all materials were exactly the same as for Experiment 1, with an exception for (a) the food items: the images included both wrapped and unwrapped food (peeled and unpeeled oranges, wrapped and unwrapped muffins, wrapped and unwrapped raisin bread rolls) and (b) the assessment of motivation influences on the activation of eating-related information (see Measures section). 

### Measures

 Similar to Experiment 1, means for each category were computed for each combination of compatible object, word, packaging (for food), and distance category were computed and natural log transformed to normalize the distribution. Furthermore, +/- 2 SD from the mean of the respective category were excluded. This exclusion led to different numbers of participants being excluded from the different analyses. Error rates, representing lacking responses to compatible trials, were computed for each category and ranged from 0,7% to 7,67%. 

 In order to examine whether motivation influences the activation of eating-related information not only hunger was assessed, but also dieting behavior and people’s reaction to food. It could be argued that dieting participants experience stronger activations of eating-related information at sight of proximal food because restraining food intake increases the saliency of external food cues [26]. Therefore, this experiment included the Restrain scale [27] measuring people’s dietary habits and the degree to which people attempt to restrict their food intake (10 items, α = .727) as well as the Power of Food scale [28] measuring the degree to which people are influenced by food (15 items, α = .866). 

## Results

### General Affordance Activation

To test the hypothesis that participants respond faster to compatible function words following objects at proximity than at distance in general q repeated measures ANOVA was conducted with object (toys vs. food), distance (proximity vs. distance) and word (compatible function vs. observation) as within-subject-factors. The results revealed a significant main effect of object *F*(1,53) = 29.591; *p* < .001; *η* ^2^_*p*_ = .358, a significant main effect of distance *F*(1,53) = 5.192; *p* < .05; *η* ^2^_*p*_ = .089, and a significant main effect of word *F*(1,53) = 36.377; *p* < .001; *η* ^2^_*p*_ = .407. Participants responded faster to food (*M* = 820.98; *SD* = 86.05) than to toys (*M* = 859.74; *SD* = 86.54); faster to proximal (*M* = 833.64; *SD* = 85.27) than to distant objects (*M* = 847.09; *SD* = 83.58), and faster to compatible function words (*M* = 808.86; *SD* = 77.66) than to observation words (*M* = 871.87; *SD* = 102.18). Moreover, a significant interaction between object and word, *F*(1,53) = 80.262; *p* < .001; *η* ^2^_*p*_ = .602 was found. For compatible function words participants responded significantly faster to food (*M* = 768.1; *SD* = 81.29) than to toys (*M* = 849.62; *SD* = 86.81), *F*(1,53) = 88.146; *p* < .001; *η* ^2^_*p*_ = .625. For observation words no such difference was observed between food (*M* = 873.86; *SD* = 111.8) and toys (*M* = 869.87; *SD* = 109.44) *F*(1,53) = 0.138; *p* = .71. Furthermore, the results showed a significant interaction between distance and word, *F*(1,53) = 4.365; *p* < .05; *η* ^2^_*p*_ = .076. Participants responded significantly faster to compatible function words at proximity (*M* = 794.53; *SD* = 87.26) than at distance (*M* = 823.19; *SD* = 79.77), *F*(1,53) = 10.409; *p* < .01; *η* ^2^_*p*_ = .164. No such distance effect was found between proximity (*M* = 872.74; *SD* = 111.34) and distance (*M* = 870.98; *SD* = 111.29) for observation words, *F*(1,53) = 0.008; *p* = .93. 

### Food

**Figure 3:**
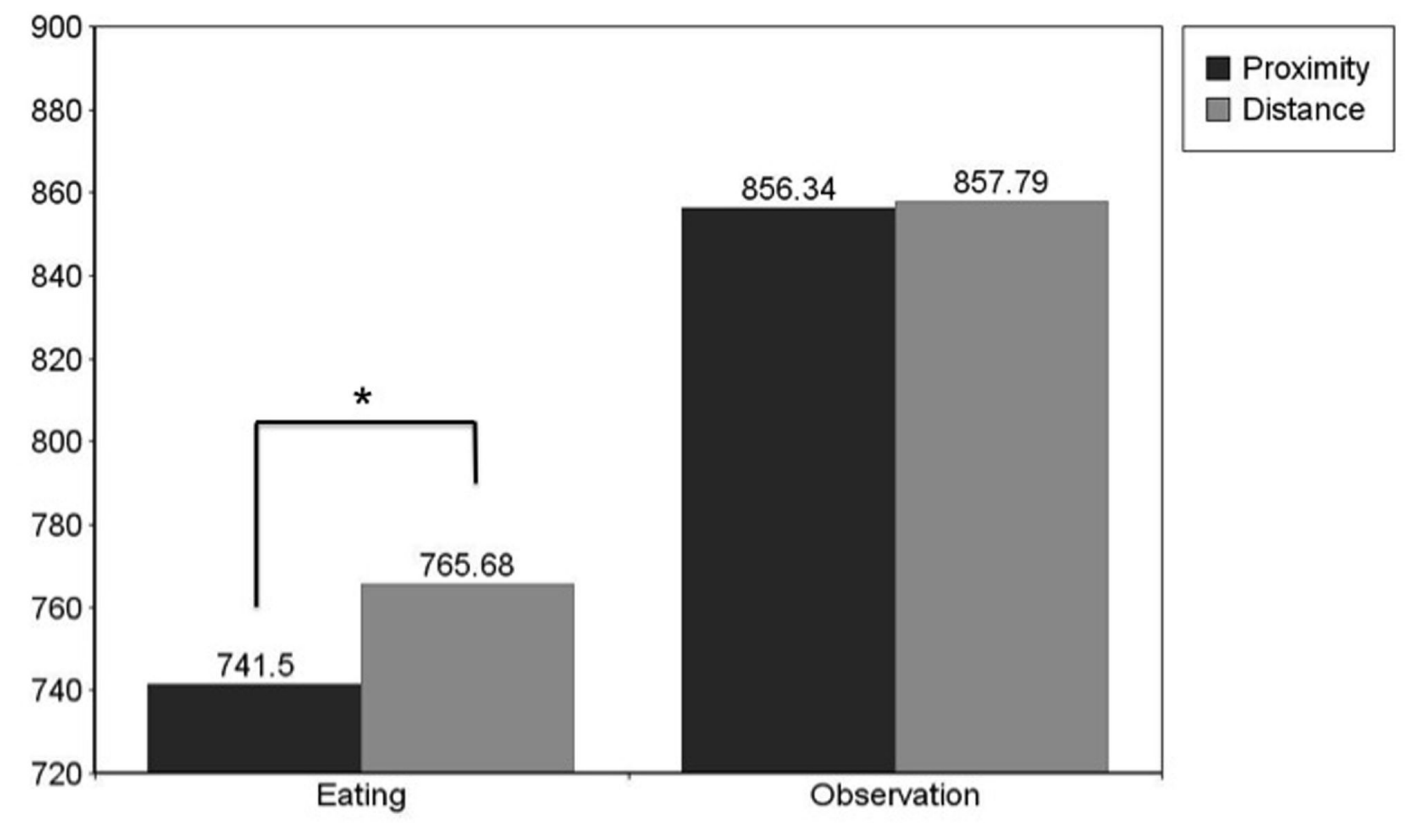
Experiment 2. **Mean Reaction Times to Unwrapped Food**. Mean reaction times to eating and observation words following unwrapped food images at proximity and at distance: Reaction times to proximal eating words are significantly faster than reaction times to distant eating words p < .05.

 To examine the second hypothesis that participants respond faster to eating words following proximal unwrapped food than distant unwrapped food and no distance effect for wrapped food and eating words planned contrasts were performed. The paired-samples t-tests revealed a significant distance effect for eating words and unwrapped food *t*(56) = -1.985; *p* (2-tailed) = .05; Cohen’s d = .24. Participants responded faster to eating words following proximal unwrapped food (*M* = 741.5; *SD* = 99.1) than distant unwrapped food (*M* = 765.68; *SD* = 98.56). This effect was not observed for eating words and wrapped food *t*(56) = -0.706; *p* (2-tailed) = .48, (proximity: *M* = 774.76; *SD* = 117.99; distance: *M* = 782.59; *SD* = 110.66); for observation words with unwrapped food *t*(56) = -0.152; *p* (2-tailed) = .88, (proximity: *M* = 856.34; *SD* = 145.26; distance: *M* = 857.79; *SD* = 137.02); or observation words and wrapped food *t*(56) = -0.709; *p* (2-tailed) = .48, (proximity: *M* = 890.18; *SD* = 176.46; distance: *M* = 868.16; *SD* = 113.02) (See Figure 3).

### Hunger and dieting

 Correlation analyses were computed between the mean scores of the Restraint scale (RS), Power of Food scale (PoF), and level of hunger and the difference score (difference between reaction times to eating words and unwrapped and wrapped food at proximity vs. distance). None of the scores was significantly related to the difference score between eating words at distance and proximity for unwrapped food. 

### Toys

**Figure 4:**
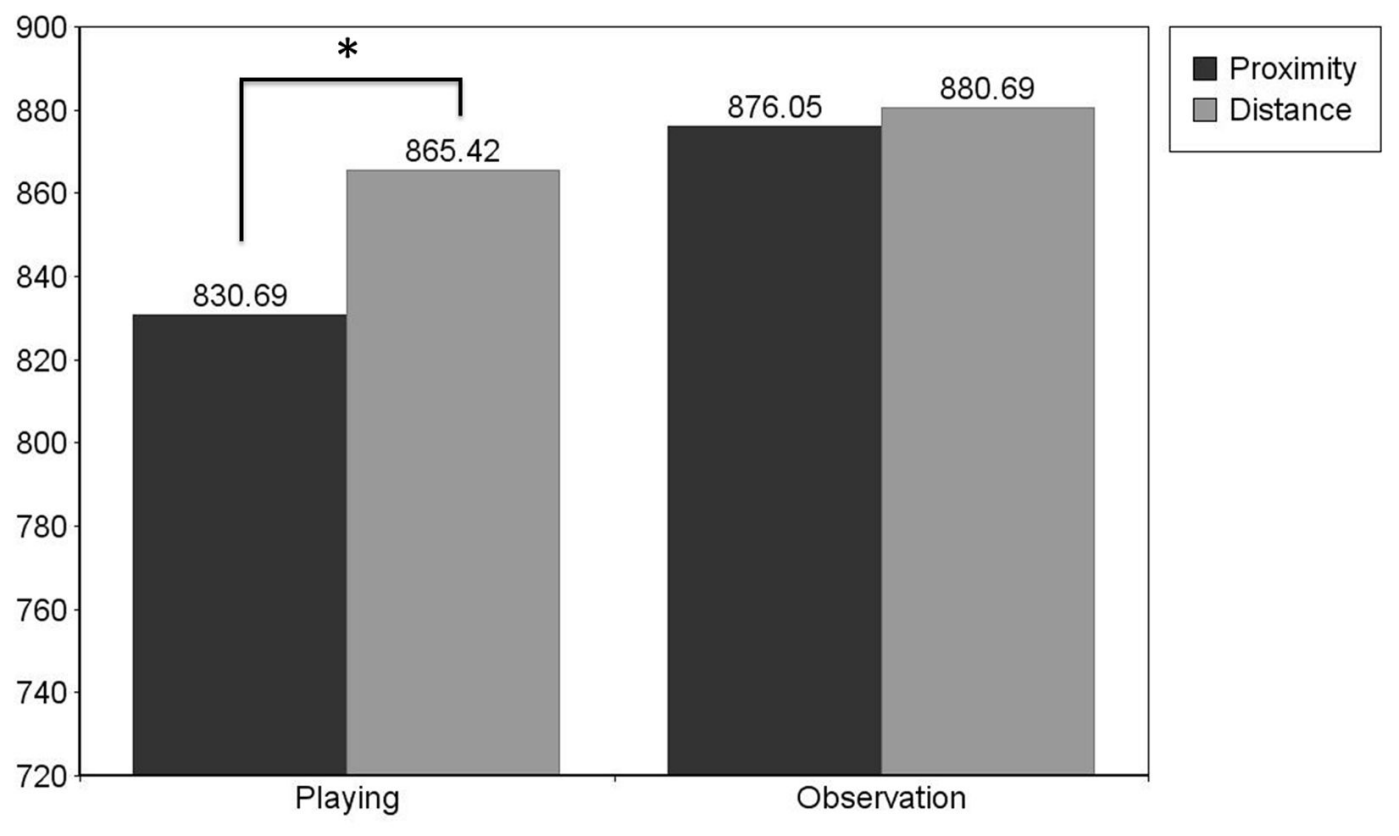
Experiment 2. **Mean Reaction Times to Toys**. Mean reaction times to playing and observation words following toy images at proximity and at distance: Reaction times to proximal playing words are significantly faster than reaction times to distant playing words p < .05.

 Testing the final hypothesis, the planned contrasts in terms of paired samples t-tests revealed a significant distance effect for playing words, *t*(57) = -3.035; *p* (2-tailed) < .01; Cohen’s d = .32. Participants reacted faster to playing words following proximal toys (*M* = 830.69; *SD* = 113.02) than distant toys (*M* = 865.42; *SD* = 106.89). There was no significant difference between responses to observation words following proximal toys (*M* = 876.05; *SD* = 144.93) and distant toys (*M* = 880.69; *SD* = 147.44, *t*(57) = -.333; *p* (2-tailed) = .74 (See Figure 4.).

## Discussion

 Experiment 2 provided additional support for the notion that proximal food and toys activate eating- and playing-related information. Participants reacted faster to compatible function words following proximal than distant objects and no distance effect was observed for observation words. As in Experiment 1, participants generally responded faster to compatible function words than observation words, faster to proximal than distant objects, and responded faster to food than to toys. 

 In initial support of the hypothesis that only actionable food activates eating-related information participants responded faster to eating words when the unwrapped food was located at proximity than at distance. This distance effect was not observed for eating responses to wrapped food or observation words. This suggests that only actionable food, in terms of distance and packaging, activates eating-related information. 

 The predicted influence of motivational state on the strength of the activation of eating-related information was not supported by the results. Consequently, we must conclude that affordances exerted by the environment are not impacted by the internal motivational state of the perceiver. 

 Finally, as predicted the distance effect for toys occurred for playing words but not for observation words. This latter result is at odds with the reversed distance effect for observation words found in Experiment 1 but in line with the hypothesis. Considering that the experiment was conducted in the same manner this result remains unexplained at this point. 

## General Discussion

 Both experiments provided support for the notion that images of proximal food and toys activate eating- and playing-related information, respectively. Participants in both experiments responded faster to compatible function words following proximal than distant objects. This distance effect occurred only for function words but not for observation words. Thus, it conforms to the expectations as function words activate information involved in the potential to interact with the object. This suggests that the accessibility of objects influences the activation of potential interactions. When interactions are possible they activate the information related to the interaction, in this case eating and playing. Therefore, eating- and playing-related information was activated upon the sight of proximal food and toys, respectively, whereas the activation of observation-related information did not depend on distance. After all, observing objects does not require objects to be proximal. Based on the rationale that only the activation of the motoric, but not the semantic system should be modulated by distance this finding indicated that proximal food and toys exert affordances for interaction. Concomitantly, the findings replicated the results of previous experiments, showing that the activation of information related to interacting with the object is modulated by the potential interactions with the object [3,14,15,29]. Objects are not merely represented in terms of their physical properties. By contrast, their representation includes the activation of information related to interactions with these objects: their affordances. Despite the original claim that artificial objects activate stronger affordances than natural objects that are represented more in terms of their sensory properties than their function [19], no structural differences in the distance effect between natural and artificial objects were discovered in the current experiments. 

 Examining the individual comparisons for toys and food similar patterns arise. For food both distance (Experiment 1 and 2) and packaging (Experiment 2) of food influenced the activation of eating-related information. Food had to be reachable and readily edible to activate eating-related information and exert eating affordances. 

 Interestingly though, participants generally responded faster to food than to toys regardless of word. This finding could be explained by the higher relevance of food compared to toys. Considering its evolutionary importance food represents a highly relevant object [20]. The main difference between the findings for food and toys remained in the reverse distance effect for toys and observation words in Experiment 1 that was not replicated in Experiment 2. At this stage this finding remains unexplained. Additional replications, for example with the inclusion of alternative artifacts, will be necessary to determine the nature of this effect. 

 In addition to replicating the influence of affordances in general, the current research extended previous findings to the realm of food and eating behavior. Showing that accessible food activates eating-related information does not conclusively show that this activation translates into eating behavior. Yet, this activation shows that whether food exerts eating affordances depends on the potential to eat it in the current context. Consequently, the finding is a first indication of the involvement of potential interaction in eating behavior that could explain the effect of increased consumption of proximal compared to distant food [1]. 

 Despite these novel findings some limitations must be addressed. Firstly, participants failed to respond to a relatively large number of compatible trials. Misses were particularly high for observation word trials. This could be related to the findings of both Experiment 1 and 2 showing that participants responded faster to function words than to observation words. While this pattern was not expected it was consistent with the findings of Costantini et al. [29]. Whereas these authors explain the difference by referring to the more functionality-based mental representation of artifacts, it could also be caused by the differences in compatibility scores between objects with function words and objects with observation words. The pre-test showed a higher compatibility for the first than the latter pair. Thus, it can reasonably be claimed that faster reaction times are related to higher compatibility. This reasoning would predict the observed findings: function words should be responded to faster than observation words. Future research should attempt to use words for observation and function that reveal similar levels of compatibility for better comparison.

 Secondly, alternative artifacts should be employed to examine the reverse distance effect for observation words. Even though this effect was only found in Experiment 1, more research needs to be conducted to understand the reversal.

 Thirdly, the design of Experiment 2 did not allow to analyze the full factorial design in one full analysis comparing the effect of distance on wrapped food, unwrapped food, and toys directly. This shortcoming occurred due to the factor packaging being nested in the food only, but not in toys. Comparing the full factorial design would have provided a stronger test of the hypotheses and future studies should ensure that such comparisons can be analyzed in an appropriate manner.

 Finally, the results revealed that eating-related information was activated upon the sight or proximal unwrapped but not wrapped food. Although this effect was expected and can be explained it is appears at odds with the fact that none of the objects were realistically actionable as they were presented on images. Previous research had shown that images of objects activate motor responses [17,19,29,30]; yet, the precise difference between the inactionability due to pictorial representation in images and the inactionability due to packaging requires further investigation. It will be necessary to examine the difference between these two kinds of inactionabilities, 

 Most importantly, future research should extend these findings to directly include the activation of motor information upon the sight of accessible food. The current results support the claim that verbal information related to the potential interaction is activated upon the sight of objects. Combining our findings with evidence from grounded cognition research showing that language comprehension and motor activation are inextricably linked [23,31] suggests that the activation of verbal information is neurologically linked to the activation of motor information. However, this was not directly tested in this research. This addition should also examine the effects of actual food at various distances and different kinds of actionability. After all, wrapped food should well activate action information involved in interaction with food; yet, this interaction should be more related to grasping and unwrapping than to immediate eating. This addition will clarify the effect of eating affordances posed by actual food in the environment on consequent eating behavior and thereby shed light on the reasons for why people experience such difficulties resisting reachable food in more naturalistic settings. Practically, the results suggest an explanation for why people overeat during meals despite being satiated and thereby contribute to understanding the detrimental effect of large portion-sizes on increased consumption. The mere perception of an unfinished portion activates eating, regardless of levels of satiation [32]. 

 Overall, the findings show that the representation of food is modulated by its accessibility. Perceiving food in the immediate proximity activates eating-related information more so than distant food. Such effects can explain people’s differential behavioral reactions to food within and food outside of reach. The findings invite future research to examine the activation of direct motor activation upon the sight of foods with different degrees of accessibility. 
